# Stability, Energetic, and Reactivity Properties of NiPd Alloy Clusters Deposited on Graphene with Defects: A Density Functional Theory Study

**DOI:** 10.3390/ma15134710

**Published:** 2022-07-05

**Authors:** Adrián Martínez-Vargas, Alfonso Vásquez-López, Carlos D. Antonio-Ruiz, Heriberto Cruz-Martínez, Dora I. Medina, Fernando Montejo-Alvaro

**Affiliations:** 1 Tecnológico Nacional de México, Instituto Tecnológico del Valle de Etla, Abasolo S/N, Barrio del Agua Buena, Santiago Suchilquitongo 68230, Oaxaca, Mexico; martinezvargasadrian@gmail.com (A.M.-V.); carlosd51912@gmail.com (C.D.A.-R.); heri1234@hotmail.com (H.C.-M.); 2 Instituto Politécnico Nacional, CIIDIR-OAXACA, Hornos Núm 1003, Col. Noche Buena, Santa Cruz Xoxocotlán 71230, Oaxaca, Mexico; bremia43@gmail.com; 3 Tecnologico de Monterrey, School of Engineering and Sciences, Atizapan de Zaragoza 52926, Estado de Mexico, Mexico

**Keywords:** binding energies, bimetallic clusters, graphene with vacancy, pyridinic N-doped graphene

## Abstract

Graphene with defects is a vital support material since it improves the catalytic activity and stability of nanoparticles. Here, a density functional theory study was conducted to investigate the stability, energy, and reactivity properties of Ni_n_Pd_n_ (n = 1–3) clusters supported on graphene with different defects (i.e., graphene with monovacancy and pyridinic N-doped graphene with one, two, and three N atoms). On the interaction between the clusters and graphene with defects, the charge was transferred from the clusters to the modified graphene, and it was observed that the binding energy between them was substantially higher than that previously reported for Pd-based clusters supported on pristine graphene. The vertical ionization potential calculated for the clusters supported on modified graphene decreased compared with that calculated for free clusters. In contrast, vertical electron affinity values for the clusters supported on graphene with defects increased compared with those calculated for free clusters. In addition, the chemical hardness calculated for the clusters supported on modified graphene was decreased compared with free clusters, suggesting that the former may exhibit higher reactivity than the latter. Therefore, it could be inferred that graphene with defects is a good support material because it enhances the stability and reactivity of the Pd-based alloy clusters supported on PNG.

## 1. Introduction

Over the last decade, bimetallic clusters or nanoparticles have received increasing attention owing to their different physical and chemical properties compared with their pure counterparts [1,2,3,4,5,6,7,8,9,10]. Due to their unique properties, these clusters can be utilized for various technological applications in the fields of catalysis, electronics, and medicine, among others [11,12,13,14]. Specifically, in the field of catalysis, the interest in bimetallic systems formed by Pd alloyed with 3d transition metals has steadily grown, largely due to their promising catalytic efficiency [15,16,17,18,19,20]. For instance, PdNi/C nanoparticles have been evaluated for the ethanol oxidation reaction, where the Pd_2_Ni_3_/C catalyst exhibits higher activity and stability in alkaline media than the Pd/C catalyst [15]. Moreover, Pd_40_Ni_60_ nanomaterials have been evaluated for methanol and ethanol oxidation in alkaline media, where the Pd_40_Ni_60_ catalyst presents a higher electrocatalytic activity than nanoporous Pd [16]. In addition, Pd, Cu, Pd_98_Cu_2_, Pd_94_Cu_6_, and Pd_86_Cu_14_ nanoparticles have been investigated for the methanol oxidation reaction [18]. Among the nanoparticles studied, the Pd_94_Cu_6_ catalyst presented the highest catalytic activity.

Nevertheless, clusters or nanoparticles tend to agglomerate, which can affect their catalytic activities and stabilities [21,22]. Consequently, the use of support materials is required to avoid this issue. In this context, graphene has proven to be a good support material due to its outstanding properties, such as a large specific surface area, corrosion resistance, excellent electrical conductivity, and good chemical stability [23,24]. However, it exhibits limited chemical reactivity [25,26]; to date, different strategies, such as defect engineering and surface functionalization, have been implemented to improve this issue [27]. Specifically, defect engineering (e.g., vacancy and doping) has proven to be an excellent method to increase the reactivity of carbon structures [27,28,29]. For example, pyridinic N-doped graphene (PNG) has proven to be a vital support material since it enhances the catalytic activity and stability of nanoparticles [30,31,32,33]. For these reasons, theoretical and experimental investigations on the reactivity and stability of nanoparticles supported on graphene with defects are important in the field of catalysis.

There are many theoretical studies on the electronic and energetic properties of monometallic clusters supported on modified graphene [34,35,36,37]; however, those on Pd alloyed with transition metals supported on modified graphene are limited, although there are some interesting studies that bear mentioning. For instance, the stability of MPd_12_ (M = Fe, Co, Ni, Cu, Zn, Pd) nanoparticles deposited on graphene with a vacancy was investigated employing the density functional theory (DFT). It was shown that the defective graphene can provide anchoring sites for these nanoparticles by forming a strong metal−graphene interaction [38]. In another study, Pd_6_Ni_4_ and Pd_4_Ni_6_ clusters supported on defective graphene have been investigated using the DFT and shown to have good stability [39]. Recently, Sánchez-Rodríguez and collaborators have studied icosahedral M@Pd_12_ (M = Fe, Co, Ni, Cu, and Pd) core-shell nanoparticles supported on PNG using the DFT [40], demonstrating that the nanoparticles have good stability and reactivity. These investigations have provided good evidence of the stability and reactivity of Pd-based bimetallic clusters deposited on graphene with defects. However, DFT computations on the stability and reactivity of Pd-based bimetallic clusters supported on graphene with defects are still required. Therefore, in this study, a DFT analysis on the stability, energy, and reactivity properties of Ni_n_Pd_n_ (n = 1−3) clusters supported on graphene with a vacancy and on PNG with one, two, and three N atoms is developed.

## 2. Computational Details

All electronic structure calculations were conducted with the DFT as implemented in the ORCA package [41]. The revised Perdew–Burke–Ernzerhof exchange-correlation functional was used in all calculations [42]. Ahlrichs basis set def2-SVP was used for the C, H, N, and O atoms [43], whereas the Pd atoms were treated using an 18-electron quasi-relativistic effective core potential [44]. The values of the convergence tolerances for geometry optimization were energy change = 5 × 10^−6^ Eh, max. gradient = 3 × 10^−4^ Eh/Bohr, rms gradient = 1 × 10^−4^ Eh/Bohr, max. displacement = 4 × 10^−3^ Bohr, and rms displacement = 2 × 10^−3^ Bohr. To investigate the stability of Ni_n_Pd_n_ (n = 1−3) clusters on PNG, most stable structures for the clusters were obtained from a previous study [2] and reoptimized using the computational details of this investigation.

**Figure 1:**
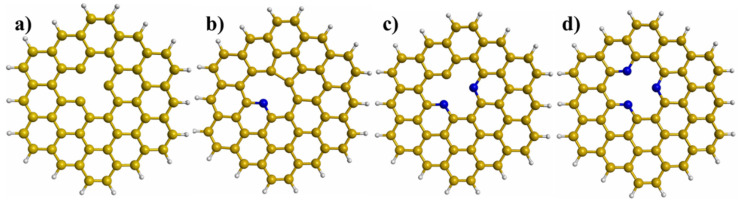
Structures of graphene with different defects: (**a**) Graphene with a vacancy, (**b**) pyridinic N-doped graphene (PNG) with an N atom, (**c**) PNG with two N atoms, and (**d**) PNG with three N atoms.

To investigate the stability, energy, and reactivity properties of Ni_n_Pd_n_ (n = 1−3) clusters deposited on graphene with different defects, circumcoronene (C_54_H_18_) was used as the graphene model. To obtain graphene with a vacancy, a C atom was removed from graphene (Figure 1a). To obtain the PNG with one, two, and three N atoms, a C atom was removed from the center of graphene to create a vacancy, and then the hanging C atoms were replaced by one, two, and three N atoms (Figure 1b–d). The binding energy (E_b_) between the bimetallic cluster and the modified graphene was calculated by the following equations:

(1) $$E_{b}=E_{\mathrm{Cluster}/\mathrm{Graphene}}-\left(E_{\mathrm{Cluster}}+E_{\mathrm{Graphene}}\right)$$

where $E_{\mathrm{Cluster}/\mathrm{Graphene}}$ is the energy of the bimetallic cluster supported on graphene with defects, $E_{\mathrm{Cluster}}$ is the energy of the bimetallic cluster, and $E_{\mathrm{Graphene}}$ is the energy of the graphene with defects.

Finally, the intermolecular interactions between the Ni_n_Pd_n_ (n = 1−3) clusters and PNG were investigated using the quantum theory of atoms in molecules implemented in the Multiwfn 3.8 program [45].

## 3. Results

### 3.1. Structures and Properties of Ni_n_Pd_n_ (n = 1−3) Clusters

**Figure 2:**
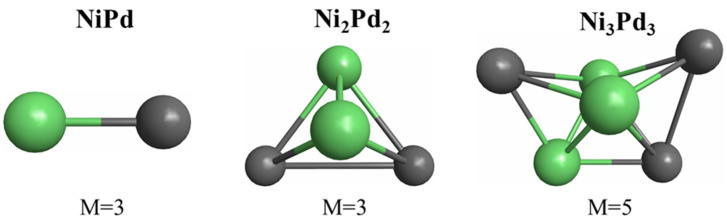
Ground-state structures of the Ni_n_Pd_n_ (n = 1−3) clusters and their respective spin multiplicities (M).

The ground-state structures of the Ni_n_Pd_n_ (n = 1−3) clusters are illustrated in Figure 2. The most stable structure of the NiPd cluster was a triplet. The ground-state structure of the Ni_2_Pd_2_ cluster was a triplet with a tetrahedral shape. Finally, the most stable structure of the Ni_3_Pd_3_ cluster was an incomplete pentagonal bi-pyramid with a spin multiplicity of five.

Different properties, e.g., the binding energy per atom (BE/n), vertical ionization potential (VIP), vertical electron affinity (VEA), and chemical hardness (η), were calculated for the ground-state cluster structures of the Ni_n_Pd_n_ (n = 1−3) clusters, Table 1. The BE/n and VEA grew monotonically when the cluster size increased. For the calculated VIP, the Ni_2_Pd_2_ cluster had the lowest value. The η was calculated from the VIP and VEA. As the cluster size increased, the η tended to decrease, which suggested that the larger clusters presented greater reactivity. The calculated properties were similar to those previously reported for these systems [2].

### 3.2. Properties of Ni_n_Pd_n_ (n = 1−3) Clusters Deposited Graphene with Different Defects

**Figure 3:**
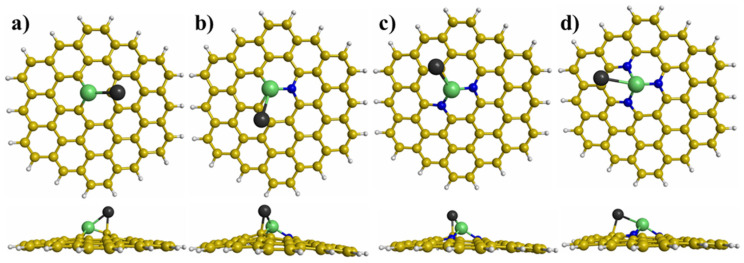
Top and side views of the most stable adsorption sites of the NiPd dimer on graphene with different defects: (**a**) graphene with a vacancy, (**b**) pyridinic N-doped graphene (PNG) with one N atom, (**c**) PNG with two N atoms, and (**d**) PNG with three N atoms.

**Figure 4:**
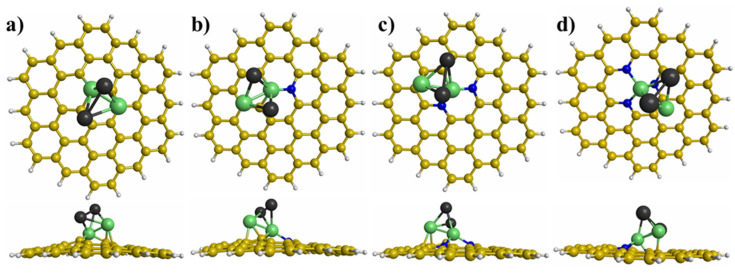
Top and side views of the most stable adsorption sites of the Ni_2_Pd_2_ cluster on graphene with different defects: (**a**) graphene with a vacancy, (**b**) pyridinic N-doped graphene (PNG) with one N atom, (**c**) PNG with two N atoms, and (**d**) PNG with three N atoms.

**Figure 5:**
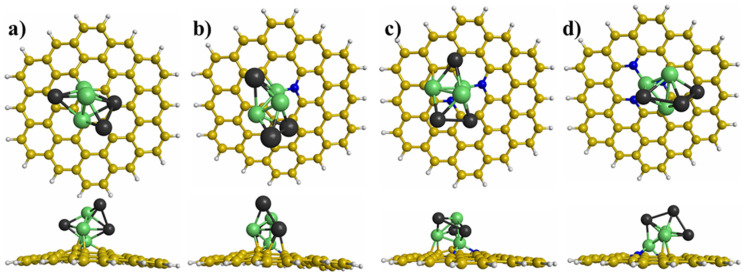
Top and side views of the most stable adsorption sites of the Ni_3_Pd_3_ cluster supported on graphene with different defects: (**a**) graphene with a vacancy, (**b**) pyridinic N-doped graphene (PNG) with one N atom, (**c**) PNG with two N atoms, and (**d**) PNG with three N atoms.

The most stable interaction between the Ni_n_Pd_n_ (n = 1−3) clusters and graphene with different defects was obtained using many initial interactions. Figure 3, Figure 4 and Figure 5 illustrate the most stable interactions between the clusters and graphene with defects. For the NiPd dimer supported on the modified graphene, the most stable interaction was with the Ni atom trapped in the vacancy of modified graphene (Figure 3). For the Ni_2_Pd_2_ cluster deposited on the graphene with defects, the most stable interaction was with two Ni atoms joined with the graphene substrates, whereby one of the atoms was anchored in the vacancy (Figure 4). Finally, for the Ni_3_Pd_3_ cluster supported on the graphene with defects, the most stable interaction was Ni atoms joined with the graphene substrates (Figure 5). For example, for the Ni_3_Pd_3_ cluster supported on PNG with three N atoms, the most stable interaction occurred with three Ni atoms, whereby one atom was anchored in the vacancy of the PNG (Figure 5).

The E_b_ between the Ni_n_Pd_n_ (n = 1−3) clusters and graphene with defects was also computed (Table 2). It was observed that E_b_ was substantially higher than that previously reported for Pd-based clusters supported on pristine graphene [46,47]. Therefore, it could be inferred that graphene with a vacancy and PNG with a different number of N atoms are good support materials for NiPd alloy clusters. It was also found that the calculated E_b_ for the Ni_n_Pd_n_ (n = 1−3) clusters deposited on graphene with a vacancy was higher compared with that calculated for the same clusters supported on PNG with a different number of N atoms. These results are similar to those reported in the literature, where it was observed that the E_b_ of Ni_n_ (n = 1−4) clusters supported on graphene with a vacancy was higher than that computed for the same clusters supported on PNG with a different number of N atoms [48,49]. After, the charge transfer between the Ni_n_Pd_n_ (n = 1−3) clusters and modified graphene was also calculated (Table 2). The results suggested that the clusters transferred a charge to graphene with defects, as these supports ended with a total positive charge greater than 0.5 *e*, which can be associated with higher E_b_, whereas for the charge transfer between the metal clusters and pristine graphene, the charge transfer is lower, which produces a low E_b_ [50]. In addition, it was observed that the Ni atoms transferred more charge than the Pd atoms to the modified graphene. This tendency is attributed to the lower electronegativity of the Ni atoms compared with Pd atoms. It was also observed that as the size of the clusters increased, the transfer of charge from the clusters to the graphene supports tended to increase.

Finally, some energetic properties were calculated (Table 2). The VIP calculated for the clusters supported on modified graphene decreased compared with that calculated for free clusters. For example, the VIPs of the NiPd dimer deposited on modified graphene ranged from 4.85 to 5.64 eV, which was less than the reported value for this dimer. Regarding the VEA, the computed values for the clusters supported on graphene with defects increased compared with those calculated for free clusters. Next, the η was calculated from the VIP and VEA. Interestingly, the η calculated for the clusters supported on modified graphene decreased compared with free clusters, suggesting that the clusters supported on modified graphene can present greater reactivity than free clusters as a small η implies an increase in the reactivity.

## 4. Conclusions

A DFT study was conducted to investigate the stability, energy, and reactivity properties of Ni_n_Pd_n_ (n = 1−3) clusters supported on graphene with defects (i.e., graphene with a vacancy and PNG with one, two, and three N atoms). The computed properties for the clusters were similar to those reported in the literature, demonstrating the reliability of the method used in this study. Regarding the stability of the Ni_n_Pd_n_ (n = 1−3) clusters supported on modified graphene, the charge was transferred from the clusters to graphene, and the E_b_ between them was substantially higher than that previously reported for Pd-based clusters supported on pristine graphene. The VIP calculated for the clusters supported on modified graphene decreased compared with that calculated for free clusters. The computed VEA for the clusters supported on modified graphene increased compared with those calculated for free clusters. The η computed for the clusters supported on modified graphene decreased compared with that calculated for free clusters, suggesting that the clusters supported on modified graphene can present greater reactivity than free clusters as a small η implies an increase in the reactivity. Therefore, it could be inferred that graphene with defects is a good support material because it enhances the stability and reactivity of the Pd-based alloy clusters supported on PNG.

## Figures and Tables

**Table 1:** Properties of Ni_n_Pd_n_ (n = 1−3) clusters.

System	BE/n	VIP (eV)	VEA (eV)	η (eV)
NiPd	1.22	7.52	0.62	3.45
Ni_2_Pd_2_	2.05	5.89	0.73	2.58
Ni_3_Pd_3_	2.47	6.20	1.57	2.32

**Table 2:** Properties of Ni_n_Pd_n_ (n = 1−3) supported on graphene with defects.

System	E_b_ (eV)	Charge (*e*)	VIP (eV)	VEA (eV)	η (eV)
NiPd/C_53_H_18_	−6.47	0.50	5.64	1.83	1.91
NiPd/C_52_H_18_N	−4.72	0.65	5.50	1.78	1.86
NiPd/C_51_H_18_N_2_	−5.26	0.66	5.15	1.67	1.74
NiPd/C_50_H_18_N_3_	−4.05	0.73	4.85	1.74	1.56
Ni_2_Pd_2_/C_53_H_18_	−6.86	0.65	5.49	1.97	1.76
Ni_2_Pd_2_/C_52_H_18_N	−4.92	0.65	5.20	2.13	1.51
Ni_2_Pd_2_/C_51_H_18_N_2_	−5.54	0.75	5.17	2.04	1.54
Ni_2_Pd_2_/C_50_H_18_N_3_	−4.65	0.73	5.37	1.99	1.69
Ni_3_Pd_3_/C_53_H_18_	−6.22	0.70	5.42	2.28	1.57
Ni_3_Pd_3_/C_52_H_18_N	−4.43	0.70	5.17	2.13	1.54
Ni_3_Pd_3_/C_51_H_18_N_2_	−4.79	0.81	4.99	1.94	1.52
Ni_3_Pd_3_/C_50_H_18_N_3_	−4.26	0.82	5.09	1.91	1.59

## Data Availability

Not applicable.
